# Cardiovascular, renal and mortality risk by the KDIGO heatmap in Japan

**DOI:** 10.1093/ckj/sfae228

**Published:** 2024-07-30

**Authors:** Shoichi Maruyama, Tetsuhiro Tanaka, Hiroki Akiyama, Mitsuru Hoshino, Shoichiro Inokuchi, Shuji Kaneko, Koji Shimamoto, Asuka Ozaki

**Affiliations:** Department of Nephrology, Nagoya University Graduate School of Medicine, Nagoya, Japan; Department of Nephrology, Tohoku University Graduate School of Medicine, Sendai, Japan; Cardiovascular, Renal and Metabolism, BioPharmaceuticals Medical, AstraZeneca, Osaka, Japan; Evidence & Observational Research, Medical, AstraZeneca, Osaka, Japan; Research and Analytics Department, Real World Data Co., Ltd, Kyoto, Japan; Research and Analytics Department, Real World Data Co., Ltd, Kyoto, Japan; Research and Analytics Department, Real World Data Co., Ltd, Kyoto, Japan; Cardiovascular, Renal and Metabolism, BioPharmaceuticals Medical, AstraZeneca, Osaka, Japan

**Keywords:** cardiovascular event, chronic kidney disease, KDIGO risk classification, observational study, renal event

## Abstract

**Background:**

This study aimed to assess the prognosis of people with chronic kidney disease (CKD) in Japan using the Kidney Disease: Improving Global Outcomes (KDIGO) heatmap.

**Methods:**

The prognoses of individuals with estimated glomerular filtration rates (eGFR) <90 mL/min/1.73 m^2^ were evaluated based on the KDIGO heatmap using an electronic medical record database in Japan. The primary outcome was major adverse cardiovascular events (MACE), a composite of myocardial infarction (MI), stroke, heart failure (HF) hospitalization and in-hospital death (referred to as MACE1). Additionally, *ad hoc* MACE2 (MI hospitalization, stroke hospitalization, HF hospitalization and in-hospital death) was examined. The secondary outcome was the renal outcome.

**Results:**

Of the 543 606 individuals included, the mean age was 61.6 ± 15.3 years, 50.1% were male and 40.9% lacked urine protein results. The risk of MACEs increased independently with both eGFR decline and increasing proteinuria from the early KDIGO stages: hazard ratios (95% confidence interval) of MACE1 and MACE2, compared with G2A1 were 1.16 (1.12–1.20) and 1.17 (1.11–1.23), respectively, for G3aA1, and 1.17 (1.12–1.21) and 1.35 (1.28–1.43), respectively, for G2A2. This increased up to 2.83 (2.54–3.15) and 3.43 (3.00–3.93), respectively, for G5A3. Risks of renal outcomes also increased with CKD progression.

**Conclusions:**

This study is the first to demonstrate the applicability of the KDIGO heatmap in assessing cardiovascular and renal risk in Japan. The risk increased from the early stages of CKD, indicating the importance of early diagnosis and intervention through appropriate testing.

KEY LEARNING POINTS **What was known:**

- The Kidney Disease: Improving Global Outcomes (KDIGO) classification was originally developed based on the risk of death, end-stage renal disease and cardiovascular death in people with chronic kidney disease (CKD).
- Despite the fact that people with CKD are at high risk for developing cardiovascular diseases, it is unclear whether the risk can be assessed with the KDIGO risk classification.
- The KDIGO risk classification predominantly draws upon evidence from Europe and the USA.

**This study adds:**

- This study demonstrated the applicability of the KDIGO risk classification in assessing the risk of cardiovascular events and showed significantly increased risk from the early stages G2A2 and G3aA1 compared with G2A1 by using a large electronic medical record database.
- Risks of cardiovascular events, renal outcomes and mortality in Japan were increased along with the KDIGO risk classification.

**Potential impact:**

- Categorizing individuals according to the KDIGO risk classification will be a great utility for better and holistic management of CKD.
- The elevated risk of events from the early stages of CKD indicates the importance of early diagnosis and therapeutic intervention through appropriate testing.

## INTRODUCTION

The prevalence of chronic kidney disease (CKD) has been increasing in recent years, affecting 850 million people worldwide and 13 million individuals in Japan. In 2017, deaths from CKD reached 1.2 million worldwide, marking a 41.5% increase since 1990 [1–3]. Therefore, CKD is predicted to become the fifth leading cause of death worldwide by 2040 [4]. CKD, together with cardiovascular complications and dialysis, is an enormous source of medical care and costs.

Although CKD is asymptomatic in its early stages, renal function declines continuously and progressively, leading to end-stage renal disease (ESRD), which necessitates renal dialysis or transplantation. The progression of CKD is also associated with an increased risk of cardiovascular events, including mortality [5, 6]. CKD and cardiovascular diseases share common risk factors, such as diabetes, obesity and hypertension [5]. The heart and kidney mutually influence each other's functions [7]. Hence, a clinical condition in one may cause a problem in the other; this is known as cardiorenal syndrome [8]. Recognizing this while managing CKD is crucial to optimal patient outcomes.

The Kidney Disease: Improving Global Outcomes (KDIGO) risk classification [9] is a matrix of estimated glomerular filtration rate (eGFR) and urinary protein level as measures of CKD status, and is widely used in the management of CKD in Japan [10]. Despite the close relationship between CKD and cardiovascular diseases, it is unclear whether the KDIGO risk classification can be directly applied to assess the risk of developing cardiovascular diseases. Moreover, most evidence used to develop the KDIGO risk classification is derived from Western countries, which differ significantly from Japan in terms of lifestyle, genetics and incidence rate of cardiovascular disease. Available evidence regarding kidney disease risk assessment is limited in Japan.

Additionally, although urine protein measurement is recommended in the management of CKD [10], previous research has demonstrated a low rate of urine protein testing in Japan [11]. Since a urine protein test is required for KDIGO risk classification, the low testing rate hinders appropriate risk evaluation. This study used a large Japanese database of routine health data in electronic medical records (EMR) to investigate the risk of major adverse cardiovascular events (MACE) based on the KDIGO risk classification in individuals with or without results of urine protein test. The findings of this study have the potential to enhance the management of CKD, ultimately leading to improved patient prognosis and health-related quality of life (HR-QoL).

## MATERIALS AND METHODS

### Study design and data source

This retrospective cohort study used the Real World Data Database (RWD-DB) maintained by the Health, Clinic, and Education Information Evaluation Institute (HCEI, Kyoto, Japan) and JMDC Inc. (Tokyo, Japan) [11]. The study was performed in accordance with ethical principles that are consistent with the Declaration of Helsinki, and the study protocol and informed consent waiver were approved by the Non-Profit Organization MINS Research Ethics Committee (approval number: MINS-REC-230220); an opt-out approach was adopted.

### Study population

Individuals who had two consecutive results of eGFR <90 mL/min/1.73 m^2^ at least 90 days apart within a 360-day period (eGFR definitive period) were included (Supplementary  data, Figs S1 and S2). The date of the second eGFR measurement that met the criteria was defined as the index date (1 January 2004–31 December 2020). JSN eGFRcr (referred to as eGFR in this study) was calculated using a previously developed formula [12]. Individuals aged 18 years or older with a minimum of 360 days of continuous enrollment before the index date (look-back period) were included. The exclusion criteria are shown in Supplementary data, Fig. S1.

### Categorizations into the KDIGO heatmap

The primary exposure was each category within the KDIGO heatmap [9]. Individuals were categorized into stages G2–G5 based on their eGFR values on the index date and into stages A1–A3 based on their urine albumin/protein levels measured closest to the index date during the eGFR definitive period. Quantitative test results and semi-quantitative results using dipstick grading (–, ±, ≥1+) were both included for the classification of the proteinuria category following this order of priority (Supplementary data, Fig. S1). Individuals who had no test data for urine protein were grouped into the “without urine protein test” group.

### Outcome definitions

The detailed definitions are provided in Supplementary data, Table S1. The primary outcome was the occurrence of MACE, which was defined as a composite of myocardial infarction (MI), stroke, heart failure (HF) hospitalization and in-hospital death (MACE1). Additionally, we established MACE2 as an *ad hoc* primary outcome designated as a composite of MI hospitalization, stroke hospitalization, HF hospitalization and in-hospital death. The secondary outcome was the renal outcome, a composite of renal replacement therapy, an eGFR decline >50%, an eGFR <15 mL/min/1.73 m^2^, a diagnosis of CKD stage 5 and in-hospital death. Both eGFR and diagnosis by the International Classification of Diseases, 10th Revision codes were used to avoid missing the event of CKD stage 5. The secondary outcome was analyzed in the population excluding individuals who received renal replacement therapy, had an eGFR <15 mL/min/1.73 m^2^, or had a diagnosis of CKD stage 5 on or before the index date. The primary and secondary composite endpoints were analyzed as time-to-first event. Individuals were followed until the occurrence of the outcome of interest, the last available data in the database, 30 September 2021, 1800 days after the index date, or death, whichever came first.

### Statistical analysis

Categorical variables are presented as numbers and percentages, while continuous data are summarized as the number of non-missing observations and presented as the mean ± standard deviation (SD). Missing values were not imputed for any of the variables.

The Kaplan–Meier estimator, with Greenwood's formula, was used to analyze the cumulative event-free survival and 95% confidence intervals (CI) of the outcomes. The crude incidence rate and 95% CI were calculated for each outcome, assuming a Poisson distribution. The hazard ratio (HR) and 95% CI of each KDIGO heatmap, for which G2A1 was used as a reference, were estimated using Cox proportional hazards models with adjustments for selected covariates (Supplementary Methods, Supplementary data, Tables S2 and S3). All analyses were performed using Python 3.7.9 (Python Software Foundation, Wilmington, DE, USA) and R version 3.6.3 (R Foundation for Statistical Computing, Vienna, Austria).

## RESULTS

### Baseline characteristics

This study included 543 606 individuals who met the eligibility criteria (Table 1 and Supplementary data, Fig. S1). Of these, 222 246 (40.9%) had no urine protein data (designated as “without urine protein test” group), and the rest of the individuals were categorized into the KDIGO heatmap according to their eGFR and urine protein test results. The mean age ± SD of the overall population was 61.6 ± 15.3 years, and 50.1% were male. The largest gap of 12 years was observed in between G2 (58.4 ± 14.7 years) and G3a (71.3 ± 11.8 years). The mean age continued to increase toward G3b (77.1 ± 10.8 years) and G4 (77.4 ± 12.1 years), and then decreased in G5 (69.5 ± 13.7 years). There were no difference in mean age from A1 (59.2 ± 14.3 years) to A2 (59.2 ± 16.0 years), and a 6-year gap to A3 (65.2 ± 16.4 years).

**Table 1:** Baseline characteristics.

						Without urine
KDIGO categories		Total	A1	A2	A3	protein test
Number of individuals included, *n* (%)	Total	543 606 (100.0)	238 826 (43.9)	52 253 (9.6)	30 281 (5.6)	222 246 (40.9)
	G2	422 376 (77.7)	199 572 (36.7)	40 979 (7.5)	16 437 (3.0)	165 388 (30.4)
	G3a	84 464 (15.5)	31 652 (5.8)	7752 (1.4)	6077 (1.1)	38 983 (7.2)
	G3b	24 589 (4.5)	6260 (1.2)	2504 (0.5)	3700 (0.7)	12 125 (2.2)
	G4	7782 (1.4)	1194 (0.2)	862 (0.2)	2458 (0.5)	3268 (0.6)
	G5	4395 (0.8)	148 (0.0)	156 (0.0)	1609 (0.3)	2482 (0.5)
eGFR (mL/min/1.73 m^2^; mean ± SD)	Total	69.2 ± 15.0	71.5 ± 12.3	69.7 ± 14.6	58.2 ± 21.8	68.0 ± 15.7
	G2	75.5 ± 8.2	75.6 ± 8.1	75.8 ± 8.1	74.7 ± 8.3	75.3 ± 8.3
	G3a	53.8 ± 4.2	54.3 ± 4.0	53.8 ± 4.2	52.9 ± 4.3	53.7 ± 4.2
	G3b	38.9 ± 4.2	39.4 ± 4.1	38.8 ± 4.3	38.0 ± 4.3	39.0 ± 4.2
	G4	23.8 ± 4.3	24.6 ± 3.9	24.0 ± 4.3	22.9 ± 4.4	24.2 ± 4.1
	G5	8.2 ± 3.5	10.5 ± 3.5	10.9 ± 2.8	9.5 ± 3.2	7.0 ± 3.2
Age (years; mean ± SD)	Total	61.6 ± 15.3	59.2 ± 14.3	59.2 ± 16.0	65.2 ± 16.4	64.3 ± 15.5
	G2	58.4 ± 14.7	56.9 ± 13.7	55.6 ± 14.9	59.5 ± 16.3	60.8 ± 15.2
	G3a	71.3 ± 11.8	69.2 ± 11.5	70.1 ± 12.8	70.8 ± 13.7	73.3 ± 11.3
	G3b	77.1 ± 10.8	76.7 ± 10.1	76.0 ± 11.7	73.5 ± 13.3	78.7 ± 9.8
	G4	77.4 ± 12.1	78.4 ± 10.6	77.1 ± 12.5	73.8 ± 13.5	79.8 ± 10.7
	G5	69.5 ± 13.7	74.6 ± 12.9	73.7 ± 13.8	70.0 ± 14.1	68.6 ± 13.3
Male gender, *n* (%)	Total	272 094 (50.1)	118 543 (49.6)	30 019 (57.4)	17 938 (59.2)	105 594 (47.5)
	G2	212 089 (50.2)	99 364 (49.8)	24 005 (58.6)	9837 (59.8)	78 883 (47.7)
	G3a	42 370 (50.2)	15 889 (50.2)	4293 (55.4)	3612 (59.4)	18 576 (47.7)
	G3b	11 515 (46.8)	2793 (44.6)	1285 (51.3)	2120 (57.3)	5317 (43.9)
	G4	3578 (46.0)	439 (36.8)	370 (42.9)	1419 (57.7)	1350 (41.3)
	G5	2542 (57.8)	58 (39.2)	66 (42.3)	950 (59.0)	1468 (59.1)

The prevalence of diabetes mellitus and hypertension increased with the progression of eGFR and proteinuria stages. The use of renin–angiotensin–aldosterone system inhibitors and diuretics trended upwards to stage G4, then plateaued, and calcium channel blockers increased up to stage G5 (Supplementary data, Table S4).

### Incidence rate of MACE

**Figure 1:**
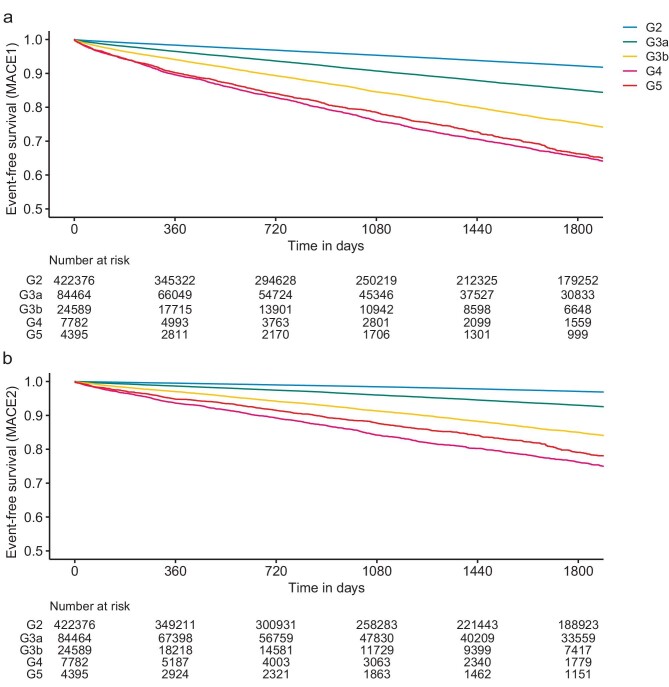
Event-free survival from MACE by KDIGO eGFR stages. (**a**) Primary outcome MACE1; (**b**) *ad hoc* primary outcome MACE2.

The event-free survival for the primary outcome MACE1 exhibited a nearly linear decrease during the 1800-day follow-up period (Fig. 1a). There was no marked difference in the event rates between the stages G4 and G5 throughout the follow-up period. Similarly, the event-free survival for *ad hoc* primary outcome MACE2 (see Materials and methods for details) also linearly decreased in all eGFR stages (Fig. 1b). In both MACE1 and MACE2, fewer events were observed in A1 (normal to mildly increased) than in A2 (moderately increased) and A3 (severely increased) (Supplementary data, Figs S3 and S4). The incidence rates of MACE1 and MACE2 were generally higher in individuals with more advanced eGFR stages, with the exception of G5 (Supplementary data, Table S5). Each component of MACE exhibited a similar trend; however, in the advanced eGFR stages, a few or no events were observed in MI hospitalization and stroke hospitalization.

### HR for MACE

The adjusted HRs, using stage G2A1 as a reference, are shown in Table 2. The risk of MACE1 increased with stage progression of CKD in both eGFR and proteinuria, and the increase was significant from the early stages G3aA1 and G2A2 with HRs of 1.16 (95% CI 1.12–1.20) and 1.17 (95% CI 1.12–1.21), respectively, to the highly advanced stage G5A3 with an HR of 2.83 (95% CI 2.54–3.15, Table 2a). The risk of MACE2 also significantly increased in the early stages of G3aA1 and G2A2 with HRs of 1.17 (95% CI 1.11–1.23) and 1.35 (95% CI 1.28–1.43), respectively (Table 2b). Similar results were obtained in two sensitivity analyses using the “strict” classification of proteinuria based on only quantitative data (Supplementary data, Table S6a and b) and in individuals having any urine protein data excluding the “without urine protein test” group (Supplementary data, Table S7a).

**Table 2:**
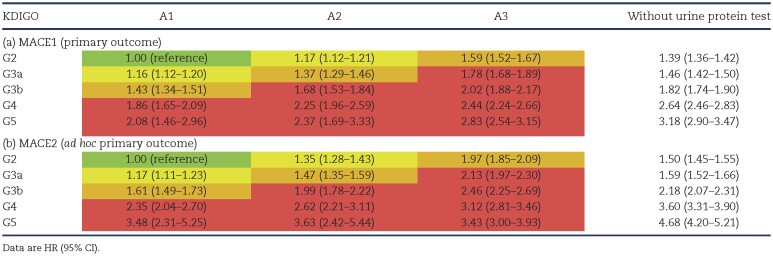
HRs for MACE in KDIGO stages.

The risk also increased in components of both MACE1 and MACE2 in the advanced stages of eGFR and proteinuria stages (Table 3). A 3- to 5-fold higher risk of HF hospitalization was observed in G4, and an approximately 4-fold higher risk of in-hospital death was observed in G5; both outcomes had higher HRs generally than MI and stroke. The risks of MI hospitalization and stroke hospitalization increased from the early stage G3aA1 with HRs of 1.39 (95% CI 1.11–1.73) and 1.23 (95% CI 1.10–1.37), respectively. The risk further increased with advancing eGFR stage among those without urine protein test. Similar results were observed in the sensitivity analysis of MACE components using the “strict” cohort (Supplementary data, Table S8).

**Table 3:** HRs for the components of MACE.

				Without urine					Without urine
KDIGO	A1	A2	A3	protein test	KDIGO	A1	A2	A3	protein test
MI^a^					MI hospitalization^b^				
G2	1.00 (reference)	1.12 (1.03–1.22)	1.42 (1.28–1.57)	1.15 (1.09–1.21)	G2	1.00 (reference)	1.35 (1.08–1.69)	1.51 (1.13–2.01)	1.26 (1.09–1.46)
G3a	1.17 (1.08–1.27)	1.32 (1.14–1.52)	1.76 (1.54–2.02)	1.25 (1.16–1.35)	G3a	1.39 (1.11–1.73)	1.50 (1.02–2.19)	1.49 (0.98–2.28)	1.48 (1.20–1.81)
G3b	1.24 (1.06–1.44)	1.55 (1.24–1.94)	1.81 (1.53–2.15)	1.58 (1.42–1.77)	G3b	0.94 (0.55–1.61)	2.08 (1.16–3.72)	2.30 (1.46–3.64)	1.97 (1.46–2.66)
G4	1.55 (1.12–2.14)	2.31 (1.65–3.23)	2.23 (1.82–2.73)	1.73 (1.40–2.12)	G4	3.25 (1.53–6.90)	2.02 (0.65–6.34)	2.32 (1.23–4.39)	2.08 (1.13–3.82)
G5	2.14 (0.89–5.14)	1.25 (0.40–3.89)	2.48 (1.94–3.16)	2.50 (2.05–3.04)	G5	NA	NA	3.62 (1.75–7.51)	5.43 (3.27–9.02)
Stroke^a^					Stroke hospitalization^b^				
G2	1.00 (reference)	1.10 (1.05–1.16)	1.44 (1.36–1.54)	1.40 (1.36–1.44)	G2	1.00 (reference)	1.26 (1.12–1.42)	1.65 (1.43–1.90)	1.45 (1.35–1.55)
G3a	1.20 (1.14–1.25)	1.38 (1.27–1.49)	1.60 (1.47–1.75)	1.48 (1.42–1.54)	G3a	1.23 (1.10–1.37)	1.38 (1.15–1.66)	1.76 (1.47–2.12)	1.50 (1.36–1.64)
G3b	1.47 (1.35–1.60)	1.51 (1.32–1.73)	1.79 (1.61–1.99)	1.61 (1.51–1.72)	G3b	1.69 (1.42–2.02)	1.60 (1.21–2.13)	1.69 (1.33–2.15)	1.70 (1.48–1.95)
G4	1.57 (1.30–1.90)	2.10 (1.70–2.60)	2.02 (1.78–2.30)	2.06 (1.83–2.31)	G4	1.62 (1.06–2.48)	1.88 (1.16–3.04)	1.82 (1.34–2.48)	2.35 (1.86–2.97)
G5	1.46 (0.81–2.65)	2.09 (1.25–3.47)	2.79 (2.40–3.23)	2.62 (2.29–3.00)	G5	0.98 (0.14–6.98)	5.09 (2.11–12.28)	2.47 (1.72–3.54)	3.60 (2.74–4.72)
Heart failure (HF) hospitalization^a^,^b^					In-hospital death^a^,^b^				
G2	1.00 (reference)	1.52 (1.33–1.72)	2.49 (2.18–2.84)	1.49 (1.38–1.61)	G2	1.00 (reference)	1.37 (1.28–1.47)	1.99 (1.84–2.15)	1.51 (1.45–1.57)
G3a	1.37 (1.22–1.53)	1.72 (1.45–2.05)	3.08 (2.65–3.58)	2.02 (1.85–2.21)	G3a	1.10 (1.04–1.17)	1.47 (1.33–1.62)	2.10 (1.91–2.30)	1.53 (1.45–1.61)
G3b	2.13 (1.84–2.47)	2.58 (2.08–3.19)	3.70 (3.12–4.39)	2.93 (2.63–3.26)	G3b	1.58 (1.44–1.74)	2.12 (1.86–2.42)	2.49 (2.23–2.77)	2.17 (2.03–2.33)
G4	3.34 (2.62–4.26)	2.93 (2.10–4.07)	5.02 (4.14–6.09)	5.01 (4.31–5.82)	G4	2.36 (1.99–2.80)	3.01 (2.47–3.68)	3.28 (2.89–3.72)	3.63 (3.28–4.01)
G5	1.98 (0.63–6.15)	1.37 (0.34–5.51)	4.79 (3.69–6.21)	4.61 (3.61–5.88)	G5	4.48 (2.85–7.05)	4.53 (2.90–7.07)	3.72 (3.17–4.37)	5.44 (4.80–6.16)

Data are HR (95% CI).

a Components of MACE1.

b Components of MACE2.

NA, not applicable due to no events observed.

### Incidence rate of renal outcome

**Figure 2:**
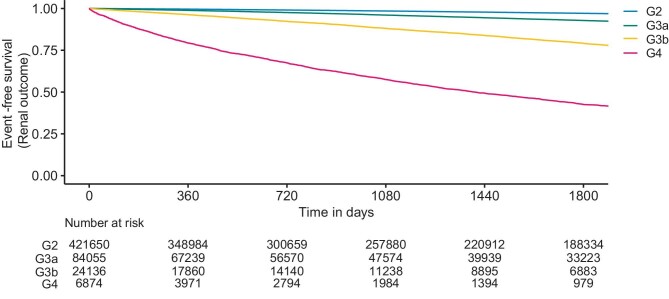
Event-free survival from renal outcome by KDIGO eGFR stages.

Event-free survival for renal outcome got lower with progression in the eGFR stage throughout the 1800-day follow-up period (Fig. 2), with a rapid and large decrease in G4 (the most advanced stage in this analysis). Across all eGFR stages, increased event rates of renal outcome were observed with the progression of the proteinuria stage (Supplementary data, Fig. S5 and Table S9).

### HR for renal outcome

The risk of renal outcome significantly increased from the early stages G3aA1 and G2A2, with HRs of 1.29 (95% CI 1.22–1.36) and 1.53 (95% CI 1.44–1.62), respectively (Table 4). A 6- to 20-fold higher risk of renal outcome was observed in stages G4A1–G4A3. The risk also increased in all renal outcome components, with worsening eGFR and proteinuria stages (Table 5). Similar results were also obtained in two sensitivity analyses using a “strict” classification (Supplementary data, Table S6c) and in individuals excluding the “without urine protein test” group (Supplementary data, Table S7b).

**Table 4:** HRs for renal outcome in KDIGO stages G2–G4.

KDIGO	A1	A2	A3	Without urine protein test
G2	1.00 (reference)	1.53 (1.44–1.62)	3.43 (3.25–3.61)	1.66 (1.60–1.71)
G3a	1.29 (1.22–1.36)	1.95 (1.81–2.11)	3.95 (3.69–4.22)	1.46 (1.42–1.50)
G3b	2.32 (2.15–2.50)	3.52 (3.19–3.88)	6.76 (6.32–7.24)	3.60 (3.41–3.80)
G4	6.84 (6.12–7.64)	10.80 (9.58–12.18)	19.96 (18.62–21.40)	10.55 (9.85–11.30)
G5	NA	NA	NA	NA

Data are HR (95% CI).

NA, not applicable.

**Table 5:** HRs for the components of renal outcome in KDIGO stages G2–G4.

KDIGO	A1	A2	A3	Without urine protein test
Renal replacement therapy				
G2	1.00 (reference)	1.46 (1.11–1.92)	9.94 (8.24–11.99)	2.13 (1.79–2.53)
G3a	2.92 (2.29–3.72)	5.60 (4.11–7.65)	20.93 (16.96–25.82)	5.76 (4.71–7.04)
G3b	10.11 (7.51–13.60)	15.77 (11.21–22.18)	56.37 (46.28–68.65)	22.64 (18.39–27.87)
G4	43.27 (30.00–62.42)	77.99 (55.96–108.70)	137.94 (113.05–168.32)	80.82 (64.08–101.94)
G5	NA	NA	NA	NA
Renal function decline >50%				
G2	1.00 (reference)	1.94 (1.77–2.14)	7.09 (6.56–7.66)	1.83 (1.71–1.95)
G3a	1.47 (1.32–1.63)	3.00 (2.62–3.44)	9.69 (8.79–10.67)	2.62 (2.40–2.85)
G3b	2.95 (2.54–3.42)	5.99 (5.06–7.09)	17.36 (15.72–19.18)	5.62 (5.07–6.23)
G4	6.35 (4.97–8.11)	13.63 (10.89–17.06)	38.35 (34.46–42.68)	13.03 (11.35–14.96)
G5	NA	NA	NA	NA
eGFR <15 mL/min/1.73 m^2^				
G2	1.00 (reference)	1.79 (1.59–2.01)	6.77 (6.17–7.44)	1.94 (1.80–2.09)
G3a	2.53 (2.28–2.80)	4.94 (4.33–5.65)	13.22 (11.93–14.66)	4.77 (4.38–5.20)
G3b	9.32 (8.32–10.44)	15.47 (13.50–17.72)	34.35 (31.22–37.79)	16.47 (15.09–17.99)
G4	44.63 (39.05–51.01)	67.14 (58.23–77.42)	122.97 (111.91–135.12)	69.47 (63.12–76.46)
G5	NA	NA	NA	NA
Diagnosis of CKD stage 5				
G2	1.00 (reference)	3.90 (2.68–5.68)	26.75 (19.92–35.93)	2.63 (1.92–3.61)
G3a	4.66 (3.11–6.97)	13.81 (8.99–21.22)	67.93 (49.88–92.51)	11.33 (8.13–15.78)
G3b	25.11 (16.53–38.13)	62.01 (41.41–92.85)	168.70 (124.80–228.05)	51.49 (37.04–71.57)
G4	133.15 (84.51–209.78)	241.34 (158.84–366.67)	470.75 (348.87–635.20)	280.17 (202.16–388.29)
G5	NA	NA	NA	NA
In-hospital death				
G2	1.00 (reference)	1.36 (1.27–1.46)	1.99 (1.84–2.14)	1.51 (1.44–1.57)
G3a	1.10 (1.03–1.17)	1.46 (1.32–1.61)	2.08 (1.90–2.29)	1.52 (1.44–1.60)
G3b	1.56 (1.42–1.71)	2.10 (1.84–2.40)	2.46 (2.21–2.75)	2.16 (2.02–2.31)
G4	2.31 (1.93–2.77)	3.18 (2.57–3.93)	3.12 (2.71–3.59)	3.59 (3.23–3.99)
G5	NA	NA	NA	NA

Data are HR (95% CI).

NA, not applicable.

## DISCUSSION

The KDIGO classification, originally developed based on the risk of death, ESRD and cardiovascular death in people with CKD [9], predominantly draws upon evidence from Europe and the USA. To the best of our knowledge, this study is the first observational study in Japan involving a substantial number of study participants who demonstrate increased risks of MACE, ESRD and mortality with progression of both eGFR and proteinuria stages based on the KDIGO heatmap. While people in G3–G4 were slightly older than those previously reported in Japanese CKD cohorts, consistent findings that mean age increased as eGFR stage progressed, with the largest gap between G2 and G3a [13–15], were provided in the present study. Furthermore, the patient distribution across eGFR stages and the prevalence of proteinuria were similar to those estimated as for all over Japan [3], supporting the notion that population examined in the present study represents Japanese clinical settings.

The risks associated with MI, stroke, HF and in-hospital death increased concomitantly with the advancement of eGFR and proteinuria stages in both MACE1 and MACE2. The increased risk was similar to that reported in the CKD Prognosis Consortium [16]. Although some uncertainty remains in MI hospitalization and stroke hospitalization due to few to no events in the advanced eGFR stages, e.g. no event in G5A1, the risk of each component showed a similar trend as the composite MACE. In particular, the adjusted HR for HF hospitalization significantly increased with decreasing eGFR and worsening urinary protein levels, suggesting a strong association between CKD progression and HF events. This is consistent with a previous report [17] showing that the development of HF in people with CKD in Japan was associated with eGFR stage progression. In the same study [17], the incidence of total mortality in stage G4 was higher than that in G5. By categorizing individuals according to their proteinuria level, the current study showed that the incidence and adjusted HR of in-hospital death increased as the eGFR stage progressed, with the highest at G5. These results demonstrate the prognostic utility of the KDIGO heatmap on MACE, emphasizing the significance of regular assessments of eGFR and urine protein for risk assessment through the KDIGO classification, in conjunction with accurate diagnosis for the effective management of CKD.

The risk of unfavorable renal outcomes also increased with CKD progression, as in the case of MACE. The magnitude of increase in risk seems somewhat small compared with that of CKD Prognosis Consortium [16]; this might be explained by residual confounding factors for HR calculation, suggesting potential underestimation of risk in the present study. Another possible explanation is differences in terms of lifestyle, genetics, patient care and medical environment between Japan and other countries included in CKD Prognosis Consortium. Nonetheless, the risk increase was inarguably high in the present study, e.g. HR for renal replacement therapy in G5A3 was 138.

The increased risk of renal events became more pronounced with the progression of proteinuria stages, as corroborated by a previous meta-analysis [16, 18]. In Japan, diabetic nephropathy is the primary cause of dialysis, accounting for approximately 40% [19]. Diabetic nephropathy is considered to develop typically with progressive albuminuria and a decline in renal function. Additionally, previous studies have confirmed that proteinuria itself is a risk factor for the progression of renal dysfunction leading to ESRD [20–22]. In the present study, HRs for renal outcome were generally higher in individuals having proteinuria within the same eGFR stage. Particularly, HR for renal replacement therapy increased up to approximately 10-fold in the early stage G2A3, demonstrating that proteinuria is associated with subsequent risk of ESRD regardless of cause, as previously reported [23]. Several randomized controlled trials have shown the association between reduced urinary albumin and reduced risk of dialysis [24–26], and CKD management guidelines recommend the measurement of urine albumin for prognostic purposes [9], highlighting the importance of interventions targeting proteinuria reduction to mitigate the need for dialysis. Given the substantial impairment of HR-QoL and the burden on caregivers, in addition to the economic burden due to lost productivity and high medical costs, many countries are striving to prevent the need for dialysis [27].

Although the importance of urinalysis is evident, challenges remain in its implementation. For example, a multicenter observational study in the USA that involved 9307 individuals with type 2 diabetes mellitus and a 15-month follow-up of their medical records reported that more than half of them were not tested for urine protein by dipstick, and awareness of CKD remained low [28]. Similarly, in the present study, about 40% of the participants had no urine protein test results. The risk in this subgroup was high, with HRs ranging from 1.39 (G2) to 3.18 (G5) for MACE1. This fact underscores the critical need for urinary protein testing to assess risk and optimize management.

The KDIGO heatmap serves not only as a tool for assessing the risk of clinical events but also as a criterion for referral to a nephrologist. Early referral has been reported to result in a better prognosis than referral at an advanced stage [29]. For the diagnosis of CKD, measurement of eGFR and urine protein is essential, especially in the early stage G2; urine protein test is important since only eGFR data are insufficient to diagnose CKD. Additionally, the progression from G2A1 to G2A2 seems to be faster than that to G3aA1: the mean age was 56.9 ± 13.7 years for G2A1, 55.6 ± 14.9 years for G2A2 and 69.2 ± 11.5 years for G3aA1, further supporting an importance of examining urine protein in stage G2. As shown in this study, a much easier dipstick test may be useful for the risk assessment of CKD. A semi-quantitative test is applicable to screening, and subsequent quantitative determination of urine protein will be preferable.

Identifying CKD is the first step to manage the disease and slow its progression [30]; however, the diagnosis rate of CKD is very low in the early stages (G2 and G3a), at approximately 10% [11, 31]. This may be due to the absence of clinical symptoms in the early stages in combination with the common misconception that declining renal function is a result of aging among older people [32, 33]. Furthermore, the slower rate of renal function decline in females may account for the lower diagnosis rate of CKD in females than in males [34–37]. Notably, the present study showed a significantly increased risk of events in the early stages (G3aA1 and G2A2) for both MACE and renal outcomes. It has also been reported that as CKD progresses, the healthcare costs required for its management increase [38]. Hence, clinical inertia may cause adverse outcomes and adverse health and economic consequences.

In recent years, sodium-glucose cotransporter 2 (SGLT2) inhibitors and mineralocorticoid receptor antagonists (MRAs) have drawn attention as promising treatments for CKD, with many studies demonstrating their renal and cardiovascular benefits [26, 39–41]. In the present study, an increased risk of developing cardiovascular disease was observed with CKD progression, indicating the importance of treatment with awareness of the cardiorenal interaction. Several studies showed that SGLT2 inhibitors and MRAs can not only prevent the progression but also improve urine protein category [42–45], which will result in reducing the risk of cardiovascular events [39, 46]. Both SGLT2 inhibitors and MRAs are also recommended in guidelines as standard treatments for HF [47–49]. Dapagliflozin, an SGLT2 inhibitor, reduces the risk of kidney failure and cardiovascular death/HF hospitalization and prolongs survival in people with CKD, regardless of the presence of type 2 diabetes [50]. The KDIGO 2024 Clinical Practice Guideline [51] recommend the use of SGLT2 inhibitors in people with CKD exhibiting proteinuria with or without diabetes. These therapeutic options are expected to slow the progression of renal function decline and prevent cardiovascular events and mortality.

This study used an EMR database. The interpretation and generalization of study results should be made cautiously because the data used in this study may not fully reflect the clinical practice in Japan, as it was sourced from contracted medical institutions (i.e. not random sampling). Additionally, diagnostic accuracy could vary among diseases, as the diagnoses in the data were derived from routinely collected health data and included diagnoses made for insurance claims; therefore, there is a possibility that they may differ from actual diagnoses. Furthermore, the data were not originally collected for research purposes; hence, there were some missing values. Specifically, urinary protein measurements were performed in individuals only when deemed necessary, and the KDIGO heatmap assessment may be affected by channeling bias. However, we adjusted for a sufficient number of covariates to mitigate this issue. Lastly, because the database was anonymized for medical institutions, no follow-up was available after individuals were transferred to other healthcare facilities, which may have caused an underestimation of the outcomes considered in this study. We assumed that eligible individuals were included in the data only once in this study; however, they may have been re-registered at other centers after transfer, potentially introducing duplications.

This study is the first to demonstrate the applicability of the KDIGO risk classification in assessing the risk of cardiovascular events and renal outcomes in Japan by using a large clinical database. The risk of events is increased from the early stages of CKD (G3aA1, G2A2), indicating the importance of early diagnosis and therapeutic intervention through appropriate testing. These insights may contribute to event reduction and HR-QoL improvement by enhancing the management of CKD in clinical practice.

## Supplementary Material

sfae228_Supplemental_Files

## Data Availability

The dataset used in the study cannot be shared publicly because the data was obtained from JMDC Inc. The data will be available on reasonable request to the corresponding author with the permission of JMDC Inc.
