# Supplementary material for: Cardiovascular, renal and mortality risk by the KDIGO heatmap in Japan
Source: Clin Kidney J. 2024 Jul 30;17(8):sfae228. doi: 10.1093/ckj/sfae228 (PMC11336683; doi:10.1093/ckj/sfae228)
Supplement: sfae228_Supplemental_Files [file sfae228_supplemental_files.zip › Maruyamaetal_Supplementary_Materials.pdf]

Supplementary material

Cardiovascular, renal and mortality risk by the KDIGO heatmap in Japan

Shoichi Maruyama, Tetsuhiro Tanaka, Hiroki Akiyama, Mitsuru Hoshino, Shoichiro Inokuchi, Shuji Kaneko, Koji Shimamoto, Asuka Ozaki

Table of Contents

Supplementary Methods 3
Supplementary Figure S1: Flowchart. 4
Supplementary Figure S2: Definition of the study population. 5
Supplementary Figure S3: Event-free survival from primary endpoint, major adverse cardiovascular event (MACE) 1 by proteinuria stages 6
Supplementary Figure S4: Event-free survival from ad hoc primary endpoint, major adverse cardiovascular event (MACE) 2 by proteinuria stages. 7
Supplementary Figure S5: Event-free survival from real outcome by proteinuria stages. 8

Supplementary Methods

Variables

For baseline characteristics and model covariates, medical history was defined as the presence of the corresponding ICD-10 codes on or before the index date. Medication usage was defined and classified according to WHO ATC codes as the presence of prescriptions during the look-back period. Medical procedures, such as emergency room visits and maintenance dialysis, were defined using the Japanese category code during the look-back period. Hospitalization was assessed using both the EMR and the Form 1 file of the Diagnosis Procedure Combination (DPC-FF1) [1], and the cause of hospitalization was derived from DPC-FF1 data during the look-back period. The detailed definitions of the baseline characteristics and model covariates are provided in Supplementary Tables S2 and S3, respectively.

The selection of covariates followed a sequential stepwise method in both forward and backward manners. Initially, the base model included age and sex as covariates. Subsequently, a set of covariates for the "cardiovascular," "renal," "other," and "drug" categories and index year (Supplementary Table S3) were sequentially incorporated into the model using the stepwise method.

Sensitivity Analysis

We performed a planned sensitivity analysis by restricting the classification of the KDIGO heatmap to quantitative urine protein test results only ("strict" cohort), and the "without quantitative urine protein test" group comprised individuals who had no quantitative results. This restriction aimed to mitigate exposure misclassification that could arise from referencing semi-quantitative results derived from dipstick grading. Additionally, ad hoc sensitivity analysis was performed by excluding individuals without urine protein test results.

Reference

1. Hayashida K, Murakami G, Matsuda S *et al.* History and profile of Diagnosis Procedure Combination (DPC): Development of a real data collection system for acute inpatient care in Japan. *J Epidemiol* 2021;**31**:1–11.

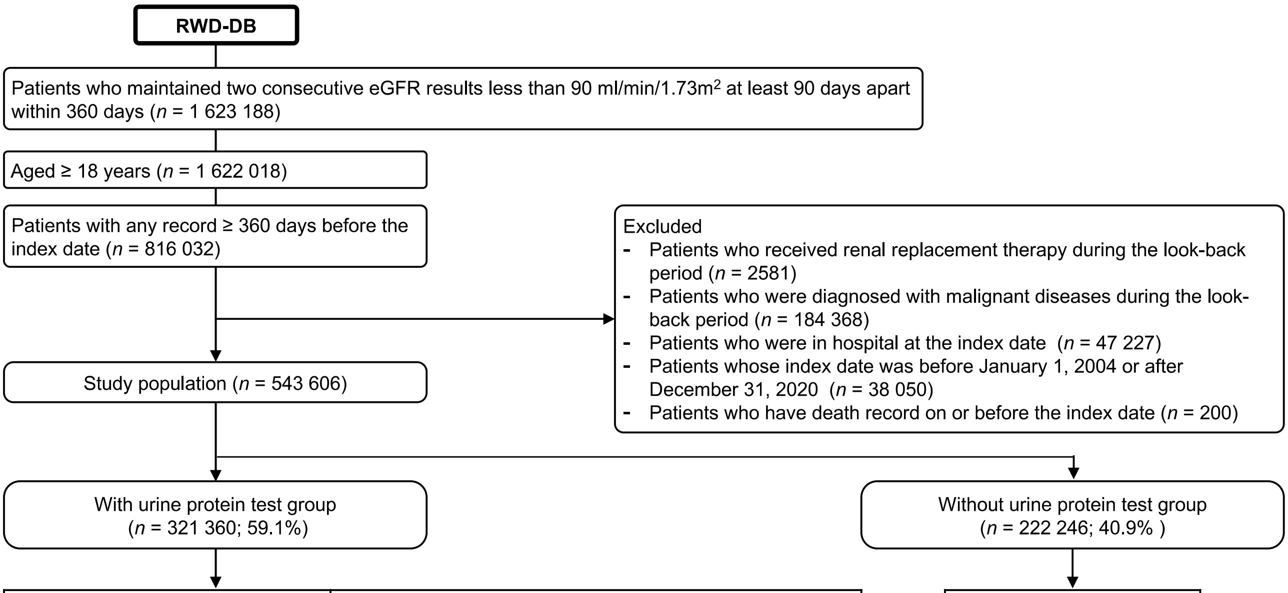

		KDIGO subgroup			Without urine protein test subgroup
Stages of albuminuria (proteinuria)		A1	A2	A3	NA
Urine albumin (mg/day, mg/gCr) semi-quantitative		<30 (–)	30≤ <300 (±)	300≤ (+)≤	NA
Urine protein (g/day, g/gCr) semi-quantitative		<0.15 (–)	0.15≤ <0.50 (±)	0.50≤ (+)≤	NA
eGFR stages (ml/min/ 1.73 m^2)	G2: eGFR 60≤ <90	199 572 (36.7%)	40 979 (7.5%)	16 437 (3.0%)	165 388 (30.4%)
	G3a: eGFR 45≤ <60	31 652 (5.8%)	7752 (1.4%)	6077 (1.1%)	38 983 (7.2%)
	G3b: eGFR 30≤ <45	6260 (1.2%)	2504 (0.5%)	3700 (0.7%)	12 125 (2.2%)
	G4: eGFR 15≤ <30	1194 (0.2%)	862 (0.2%)	2458 (0.5%)	3268 (0.6%)
	G5: eGFR <15	148 (<0.1%)	156 (<0.1%)	1609 (0.3%)	2482 (0.5%)

Supplementary Figure S1: Flowchart.

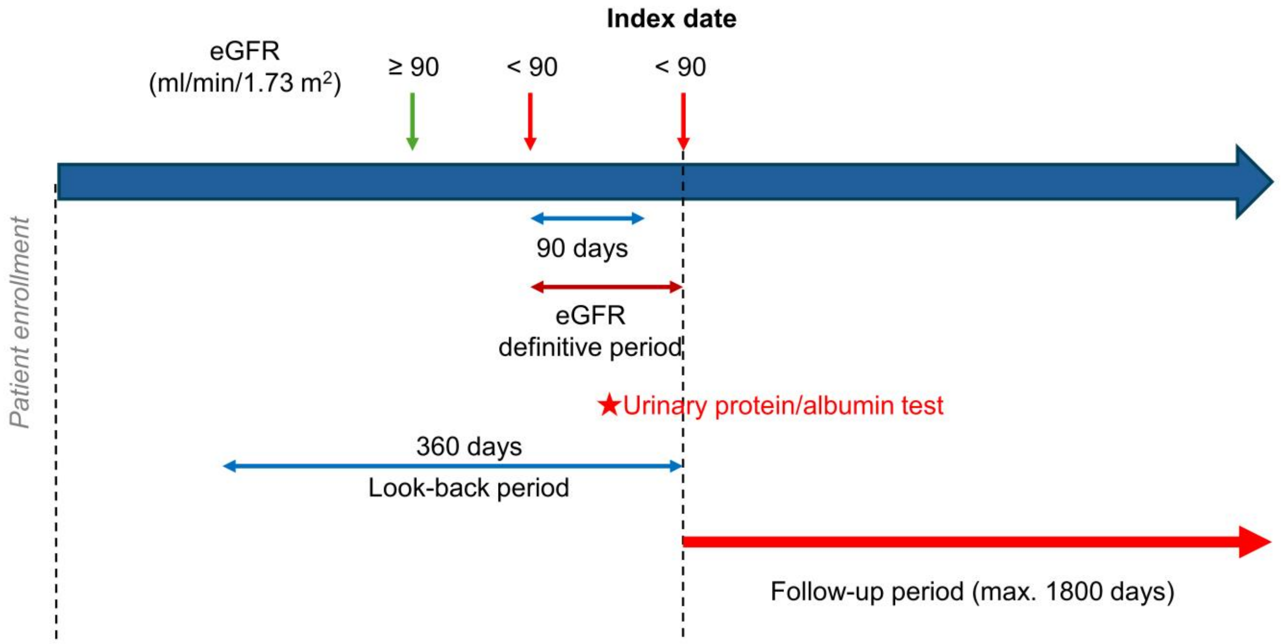

Supplementary Figure S2: Definition of the study population.

When an individual had two or more candidates for the index date, the earlier date was used. In cases where multiple eGFR values were obtained on the same day, the lowest value was used. If an individual had two or more urine test results on the same day, the highest value was used.

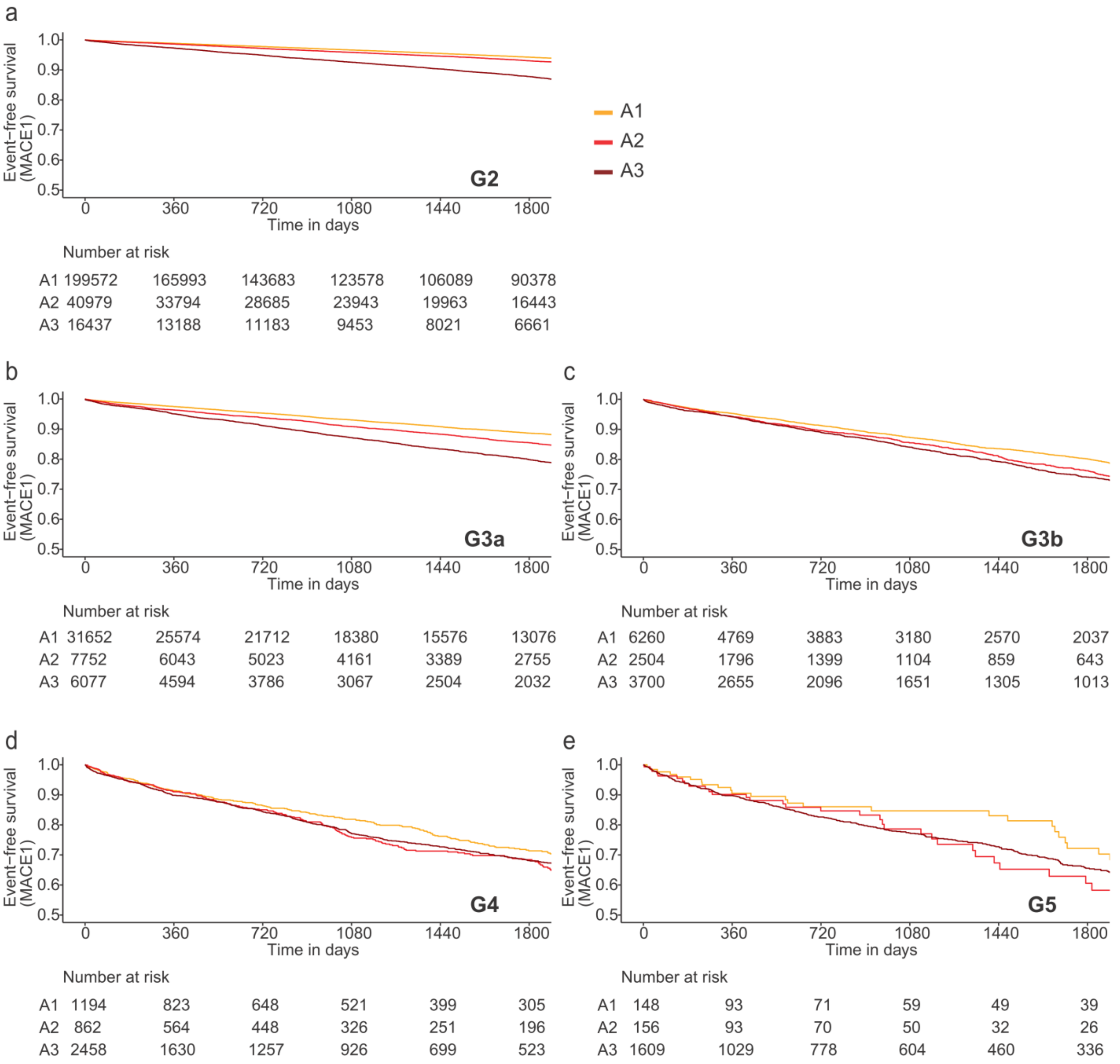

Supplementary Figure S3: Event-free survival from primary endpoint, major adverse cardiovascular event (MACE) 1 by proteinuria stages.

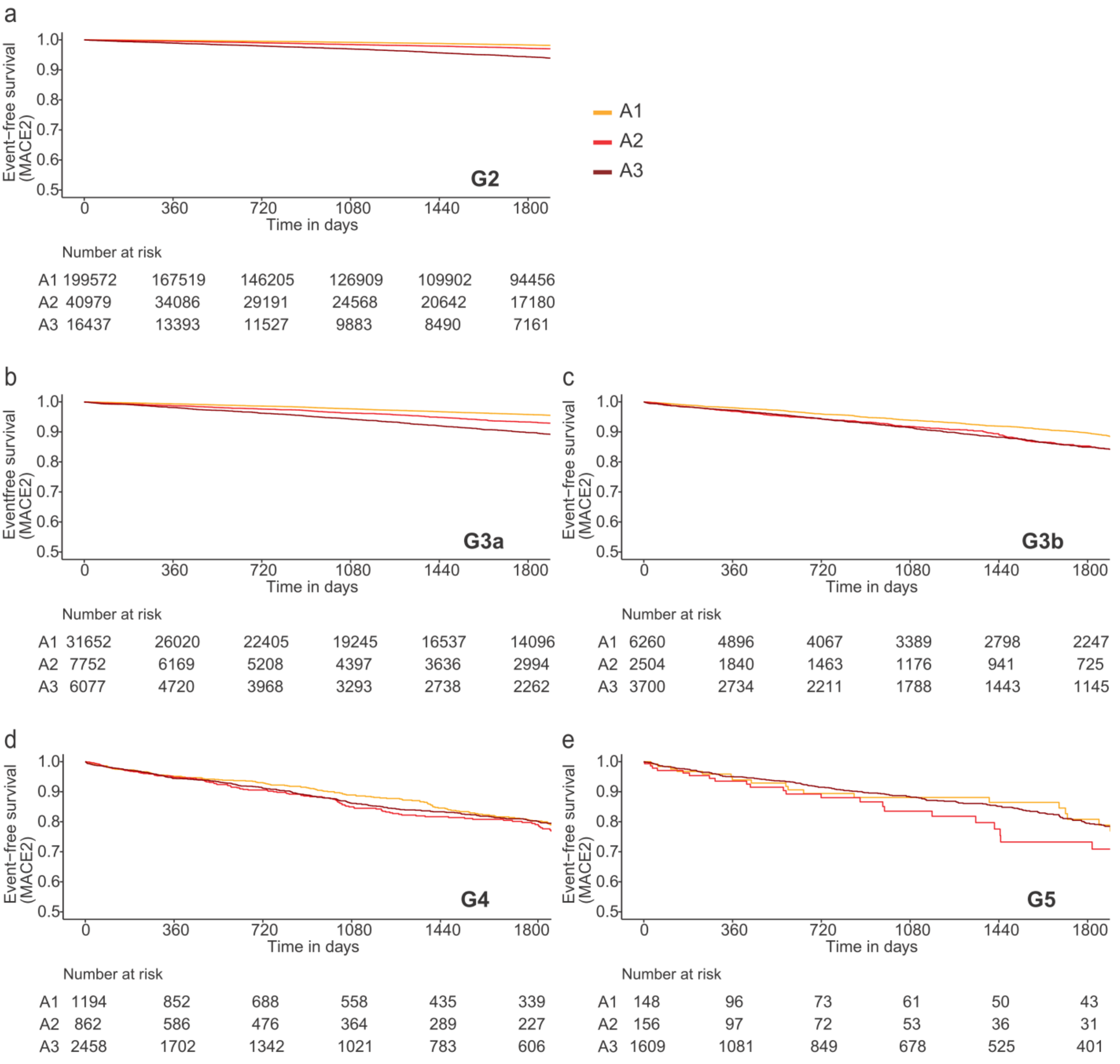

Supplementary Figure S4: Event-free survival from ad hoc primary endpoint, major adverse cardiovascular event (MACE) 2 by proteinuria stages.

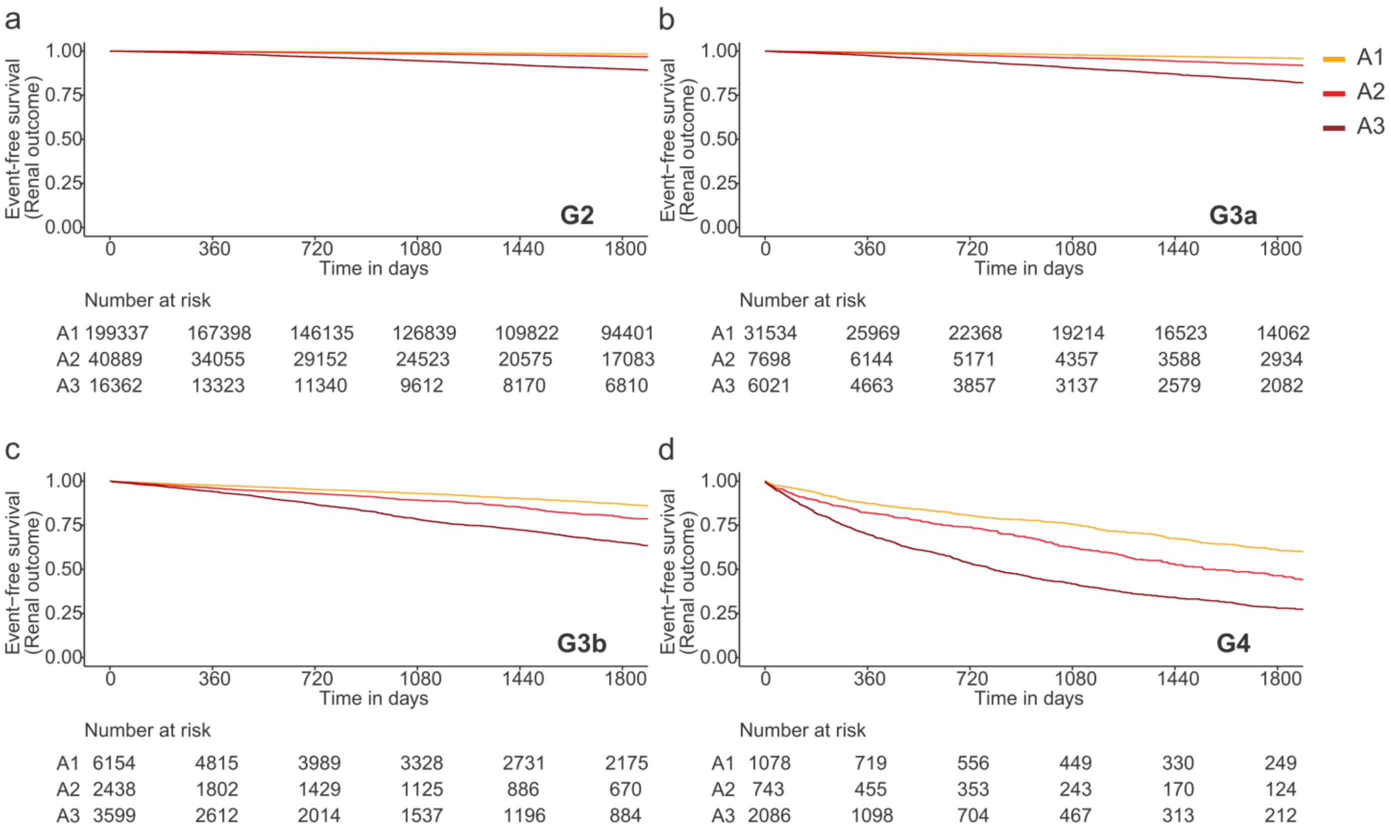

Supplementary Figure S5: Event-free survival from real outcome by proteinuria stages.
